# Capturing Emotions Induced by Fragrances in Saliva: Objective Emotional Assessment Based on Molecular Biomarker Profiles

**DOI:** 10.3390/bios16020081

**Published:** 2026-01-28

**Authors:** Laurence Molina, Francisco Santos Schneider, Malik Kahli, Alimata Ouedraogo, Mellis Alali, Agnés Almosnino, Julie Baptiste, Jeremy Boulestreau, Martin Davy, Juliette Houot-Cernettig, Telma Mountou, Marine Quenot, Elodie Simphor, Victor Petit, Franck Molina

**Affiliations:** 1 Sys2Diag UMR9005 CNRS/ALCEN, Biological Complex System Modeling and Engineering for Diagnosis, 1682 rue de la Valsière CS 61003, 34184 Montpellier Cedex 4, France; laurence.molina@sys2diag.cnrs.fr (L.M.); francisco.santos-schneider@sys2diag.cnrs.fr (F.S.S.); malik.kahli@sys2diag.cnrs.fr (M.K.); vpetit@skillcell-alcen.com (V.P.); 2 SkillCell, 1682 rue de la Valsière CS 61003, 34184 Montpellier Cedex 4, France

**Keywords:** emotional assessment, emotion molecular biomarkers, olfactive stimulation, salivary biomarker assay, cortisol, DHEA, alpha-amylase, oxytocin

## Abstract

In this study, we describe a non-invasive approach to objectively assess fragrance-induced emotions using multiplex salivary biomarker profiling. Traditional self-reports, physiological monitoring, and neuroimaging remain limited by subjectivity, invasiveness, or poor temporal resolution. Saliva offers an advantageous alternative, reflecting rapid neuroendocrine changes linked to emotional states. We combined four key salivary biomarkers, cortisol, alpha-amylase, dehydroepiandrosterone, and oxytocin, to capture multidimensional emotional responses. Two clinical studies (n = 30, n = 63) and one user study (n = 80) exposed volunteers to six fragrances, with saliva collected before and 5 and 20 min after olfactory stimulation. Subjective emotional ratings were also obtained through questionnaires or an implicit approach. Rigorous analytical validation accounted for circadian variation and sample stability. Biomarker patterns revealed fragrance-induced emotional profiles, highlighting subgroups of participants whose biomarker dynamics correlated with particular emotional states. Increased oxytocin and decreased cortisol levels aligned with happiness and relaxation; in comparison, distinct biomarker combinations were associated with confidence or dynamism. Classification and Regression Trees (CART) analysis results demonstrated high sensitivity for detecting these profiles. Validation in an independent cohort using an implicit association test confirmed concordance between molecular profiles and behavioral measures, underscoring the robustness of this method. Our findings establish salivary biomarker profiling as an objective tool for decoding real-time emotional responses. Beyond advancing affective neuroscience, this approach holds translational potential in personalized fragrance design, sensory marketing, and therapeutic applications for stress-related disorders.

## 1. Introduction

Fragrances have long been recognized for their capacity to elicit strong emotional responses, ranging from pleasure and nostalgia to aversion and heightened alertness. Quantifying these effects is marked by a critical challenge: the act of measurement itself should not disrupt the emotional state being assessed. Traditional methods, such as self-report questionnaires, behavioral observation, electroencephalography (EEG), functional magnetic resonance imaging (fMRI), or peripheral physiological monitoring (e.g., electrodermal activity and heart rate variability), have contributed valuable insights to the field but are often intrusive, limited in temporal resolution, or influenced by cognitive biases [1,2,3]. At a biological level, olfactory stimuli raise complex responses involving both central and peripheral systems. Signals from olfactory receptor neurons bypass the thalamus and project directly to the primary olfactory cortex and limbic structures, notably the amygdala, hippocampus, and orbitofrontal cortex [4,5]. These regions mediate both odor perception and emotional salience, engaging the hypothalamic–pituitary–adrenal (HPA) axis and the autonomic nervous system (ANS) to produce measurable changes in peripheral physiology [6,7]. Despite these well-established links, there is currently no objective molecular method able to characterize emotional responses to olfactory stimulation in peripheral biofluids in real time.

The amygdala responds rapidly to odor valence, even without conscious awareness [5]. Pleasant and unpleasant odors elicit distinct activation patterns in the amygdala and orbitofrontal cortex [8], with hippocampal activity underlying the “Proust phenomenon” of odor-evoked autobiographical memory [9]. These central processes are tightly coupled to peripheral outputs: stress-related odors can increase alpha amylase release [10], and pleasant odors can reduce cortisol levels [7] and perceived level of pain [11]. Moreover, certain odorants modulate sympathetic activity within seconds [6]. Such pathways highlight the integration between central detection, valence coding, and peripheral effectors (including hormones, neuropeptides, and enzymes), many of which can be detected in accessible biofluids such as saliva.

Existing tools for assessing emotion include self-report, physiological monitoring, and neuroimaging. Self-report measures capture the subjective experience but are vulnerable to introspection errors and social desirability bias [12]. Physiological indices such as heart rate or skin conductance provide objective autonomic data yet lack the molecular specificity needed to dissect the biochemical mechanisms of emotion [13]. Neuroimaging and electrophysiological techniques offer high neural resolution but are costly, stationary, and often intrusive [14,15]. While valuable, these methods do not deliver a direct molecular readout of the peripheral physiological signature of emotion, particularly in response to sensory stimulation, and many are unsuitable for real-time or repeated use in naturalistic settings [16].

Since humans can dynamically integrate environmental exposures (the exposome), they function as sensors, initiating rapid responses through both central and peripheral mechanisms [17]. Consequently, a relevant approach is to measure emotions through biofluids that capture rapid physiological dynamics. Such a matrix must possess rapid turnover and be sensitive enough to capture moment-to-moment changes, be able to reflect central nervous system (CNS)-regulated neuroendocrine activity, and allow repeated non-invasive sampling without intervention by medical personnel [18,19,20]. Saliva fulfills all of these criteria; it contains hormones, neuropeptides, enzymes, and metabolites whose concentrations vary over minutes to hours and are influenced by the CNS and autonomic activity [21]. Its proximity to the CNS and the permeability of the blood–brain barrier allow it to reflect acute neuroendocrine changes, and its ease of collection minimizes emotional disturbance during sampling.

A growing body of empirical research links salivary biomarkers to emotional and stress responses, each reflecting a distinct physiological pathway. Cortisol (CRT), the end product of HPA-axis activation, typically rises within minutes after stress onset and modulates emotional memory and vigilance [2,22]. Odor-induced stressors have been shown to modulate salivary cortisol [7]. Levels of salivary alpha-amylase (sAA), a surrogate marker of sympathetic activation, rise within minutes of emotional arousal, offering high temporal resolution for detecting acute changes in stress and alertness [18,23]. Dehydroepiandrosterone (DHEA), produced by the adrenal cortex, exerts neuroprotective and anti-glucocorticoid effects and has been associated with emotional resilience [24,25]. Oxytocin (OXT), a neuropeptide involved in social bonding and affect regulation, can modulate amygdala reactivity to emotional stimuli [26], and changes in salivary OXT have been reported following social and sensory stimulation [27,28]. Together, these biomarkers (BMs) enable multidimensional profiling of emotional states, spanning stress, arousal, resilience, and social-affiliative processes.

To date, the use of up to two salivary biomarkers in combination to profile emotional responses to odors has been limited. Most authors have focused on stress paradigms [3,6], leaving a gap in understanding how positive, neutral, or socially relevant odors are represented in peripheral molecular signatures. Integrating larger multiple biomarkers should enable differentiation between emotional states that share similar valence but differ in arousal or in their relevance to social contexts.

In this work, we showed that saliva could capture the peripheral molecular signature of emotion in real time during olfactory stimulation. Simultaneous measurement of four biomarkers, namely, CRT, sAA, DHEA, and OXT, can reflect a substantial part of the complexity of human emotional responses. We further hypothesize that distinct emotional states (or simplified valence/arousal profiles) can be mapped to reproducible biomarker variation profiles. This approach demonstrates the capacity of a non-invasive, scalable method for objective emotional assessment in both research and applied contexts.

## 2. Materials and Methods

*Participants:* The study was performed in accordance with applicable guidelines and regulations and informed consent was obtained from all participants prior to their inclusion. The study was approved by a French national ethics committee (CPP-SUD EST I) on October 2022 (22.03698.000189). The panel consisted of healthy volunteers aged 18–60 years, either naïve or trained in olfactory stimulation.

*Saliva collection and storage:* Participants were required to refrain from eating, drinking or smoking for at least 30 min prior to saliva collection. Saliva samples were collected via passive drooling into sterile polystyrene tubes (minimum volume of 2 mL) obtained for each sample. Visual inspection confirmed the absence of blood contamination. Immediately after collection, the samples were placed on ice and processed on the same day. After treatment, samples were used immediately or stored at −80 °C.

**Figure 1:**
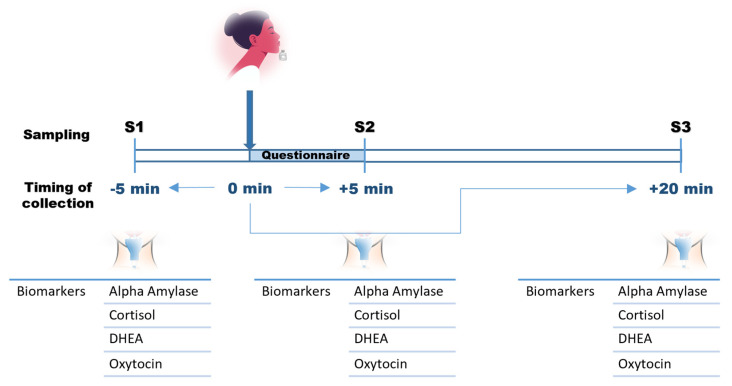
Design of the clinical research studies. The subjects’ participation consisted of (1) a 5 min sit-down rest before the first sampling of saliva (5 min before olfactory stimulation), (2) smelling the fragrance and responding to the questionnaire, and (3) a second and (4) third saliva sampling five and twenty minutes after smelling the fragrance, respectively.

*Clinical studies:* We designed and conducted two clinical studies and a consumer test, each involving a separate group of volunteer subjects (n = 30, n = 63, n = 80, respectively). During the clinical research studies (n = 30 and n = 63), participants smelled one fragrance per day, in the morning, and the order of the fragrances was randomized for all participants. Saliva samples were collected at three different time points during the panel: 5 min before stimulation (S1) and 5 min (S2) and 20 min (S3) after stimulation by a specific olfactory fragrance (Figure 1). Six different fragrances were studied (two fragrances in the first study (F0–F1) and four in the second (F2–F5)). Participants completed a questionnaire about their emotional responses immediately after stimulation and before the S2 sample was collected.

During the implicit association test, the panelists (n = 80) smelled the four fragrances, F2–F5. Individual emotional evaluations were performed using a combination of a questionnaire (six to nine emotions assessed) and the aforementioned test.

*Biomarker measurement in saliva:* Salivary cortisol, DHEA, and oxytocin were assayed using commercial competitive enzyme-linked immunosorbent assays purchased from Cayman Chemical (MI, USA), Tecan (Hamburg, Germany), and Enzo Life Sciences (NY, USA), respectively. Salivary alpha-amylase was measured using an enzymatic assay purchased from IBL International (Tecan, Hamburg, Germany). Saliva samples were analyzed immediately after collection.

*Emotional assessment by panelists:* For each of the olfactory stimulations, separate emotional assessments were performed using a questionnaire. The questionnaire evaluated certain parameters related to fragrances, such as intensity and valuation, in addition to the emotions aroused, including “confident”, “sensual”, “happy”, “dynamised”, “relaxed”, and “comforted”. For the first clinical study, three additional emotions were assessed: “elegant”, “addict”, and “unique”. Participants classified their emotions and feelings into levels ranging from 0 to 2 (0 lowest valence, 2 highest valence).

An independent implicit association test was performed on a separate set of panelists (N = 80; 50% men; 50% women) with eight fragrances. Participants who did not consider fragrances important and did not use them regularly were removed from the panel. Subjects were instructed to press the space bar on the computer if the emotional attribute administered matched their emotional state when they smelled the fragrance. Only four fragrances were included in the main clinical study.

*Data analyses and statistics:* Differences between quantitative variables were assessed with the Wilcoxon rank sum test, Kruskal–Wallis test, or ANOVA. We corrected for multiple hypotheses with the Benjamini–Hochberg test. PCA was performed using the R function prcomp in the stats library [29]. To perform Linear Discriminant Analysis (LDA), a supervised method, a 2-fold cross-validation was employed to compute accuracies; a mean of 100 repeats was obtained. LDA was performed with the R function lda of the MASS_7.3-60 library [29]. K-means and hierarchical k-means (hk-means) were performed with the R function k-means in the stats library and hk-means in the factoextra_1.0.7 library. The hierarchical clustering step of the hk-means was performed using the Euclidian distance to measure the distance between individuals, and Ward’s minimum variance method combined with the squares Euclidian distance was used as a linkage method. The formula of Calinski & Harabasz in the function Nbclust() in the R package Nbclust (v4.3.2; R Core Team 2023) was used to optimize the cluster numbers. Radar chart graphs were obtained with the function radarchart of the fmsb_0.7.6 R library. The CART algorithm was employed using discrete variables of S2/S1 ratios of biomarkers and executed using the rpart (version 4.1.24) and rpart.plot (version 3.1.2) libraries in R software. Graphs were generated using the Python libraries matplotlib (version 3.10.0), pandas (version 2.2.3), and numpy (version 2.0.2).

## 3. Results

### 3.1. Reference Testing and Analytical Validation of Salivary Biomarkers: Stability, Robustness, and Circadian Considerations

To ensure the robustness and coverage of our results, we cautiously compared the measurement of four salivary biomarkers (CRT, sAA, DHEA, and OXT) by comparing at least two commercial assay kits for each biomarker based on rigorous criteria (e.g., linearity, dynamic range, and assay reproducibility), with the best-performing kits selected. To ensure effective saliva sampling, correct transport, and the reliability of the biomarker measurements after storage, we evaluated the stability of the samples. For this purpose, salivary biomarkers were tested under multiple temperature storage conditions (−20 °C, 4 °C, room temperature (RT), 37 °C, 42 °C, and 60 °C for 48 h) (Supplementary Figure S1). DHEA and CRT remained relatively stable across temperatures of −20 °C, +4 °C, RT, 37 °C, and 42 °C (CRT); in comparison, sAA remained stable only up to RT, and OXT showed poor stability, even at −20 °C. Based on these results, all biomarkers were analyzed on the day of sample collection. The inter-day and individual variation and the impact of circadian rhythm were assessed in samples collected throughout the day (Supplementary Figure S2). Salivary CRT, DHEA, and OXT concentrations were higher in the early morning (9 a.m.), declining progressively through the day, with a modest effect for OXT; in comparison, sAA activity exhibited the opposite pattern, markedly increasing after midday. These findings are consistent with previously published data [30,31]. As the olfactory stimulation during the clinical study lasted approximately 30 min and short-term biomarker variations were minimal, approximately 6% over 30 min, we therefore chose to collect samples in the morning.

### 3.2. Olfactory-Induced Emotions Rapidly Impact Salivary Biomarker Levels

**Figure 2:**
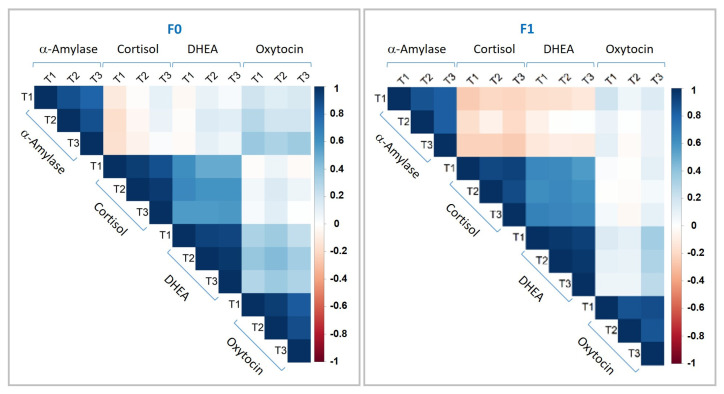
Correlation heatmaps of variances between the four biomarkers for F0 and F1 fragrances. T1: collection of saliva samples 5 min before stimulation; T2: collection of saliva samples 5 min after stimulation; T3: collection of saliva samples 20 min after stimulation. The gradient color represents the degree of correlation between two measurements: 1 = maximum correlation; −1 = maximum anti-correlation. Correlations were calculated using the Spearman method.

We examined whether olfactory stimulation modulates salivary biomarkers and whether changes depend on the odorant, its support, or the test procedure itself. A pilot study was conducted including 30 participants, with repeated saliva sampling to capture biomarker kinetics following exposure to either the fragranceless support (F0) or a fragrance (F1) stimulus (Figure 1). Across conditions, cortisol and DHEA were partially correlated after F1 or F0 stimulation (Figure 2). In contrast, sAA showed a negative correlation with cortisol and DHEA, but only in response to the fragrance F1. OXT displayed a distinct pattern, diverging from the other BMs. These differences between the F0 and F1 fragrances were consistent across the three sampling time-points (S1, S2, and S3), supporting the decision to retain all four biomarkers to capture the emotional complexity through a multidimensional molecular signature. The baseline of observed biomarker concentrations (S1, before stimulation) revealed relatively broad inter-individual variability (e.g., sAA: 176.1 ± 6.5 U/mL; CORT: 2076.2 ± 103.2 pg/mL; DHEA: 427.2 ± 30.0 pg/mL; OXT: 171.1 ± 17.6 pg/mL). To account for the above and differing measurement units, biomarker values are expressed as post-stimulation ratios relative to baseline value (S2/S1 and S3/S1), enabling comparisons on the same scale.

Analyses focusing on individual responses to fragrances revealed high inter-individual variability. More than half of the subjects exposed to the F0 fragrance reported little-to-no emotion in the questionnaire in parallel to minimal biomarker fluctuations (e.g., subjects E-010 and E-024 for the F0 fragrance; Supplementary Figure S3). In contrast, only three subjects failed to report an emotional response to F1. Several individuals (e.g., E004, E007, and E010) showed marked biomarker shifts within 5 min of odor exposure and also exhibited a highlighted emotional response. These data suggest that sensitivity to olfactory stimuli clearly varies across individuals and can be detected rapidly at the biomarker level. Through unsupervised k-means clustering analysis of biomarker profiles, we identified subgroups of participants exposed to the F1 fragrance, whose biomarker dynamics aligned with distinct emotional responses (in particular for clusters 2, 4, and 5 (Supplementary Figure S4)). Our exploratory analysis results demonstrate that multivariate biomarker patterns can differentiate both stimulus-related effects and inter-individual variability in fragrance-induced emotions.

### 3.3. Identification of Salivary Biomarker Profiles Related to Olfactory Emotion-Induced Responses

To extend our proof-of-concept findings, we conducted a larger study (n = 63) using four different fragrances (F2–F5). Four participants smelled two or three fragrances only, leaving a total n = 59 participants being stimulated by all four fragrances, resulting in 708 saliva samples across 236 participant–fragrance combinations and three sampling points (S1–S3; Figure 1). The biomarkers were measured robustly, with mean coefficients of variation <9% for each BM.

Across the full cohort, partial weak correlations once again emerged between DHEA and cortisol; in comparison, the other biomarkers varied independently (Supplementary Figure S5). This independency confirms the complementary contribution of each BM to the molecular signature of emotions as observed in the first clinical study.

### 3.4. Individual Analyses of Fragrance-Induced Molecular Profiles Reveal a Clear Association with Emotional Response

**Figure 3:**
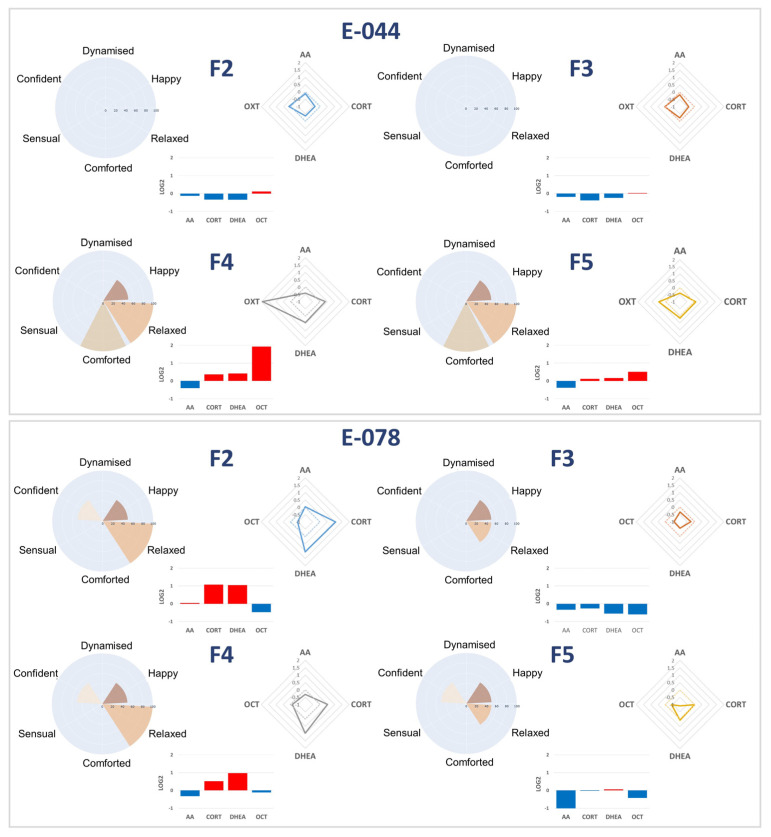
Individual case studies of emotional responses and biomarker ratio (S2/S1) profiles by fragrance for participants E-044 and E-078. The emotional response profiles are represented by radar plots (**left**) that described the valence for each emotion assigned by individual participants. Log2 mean biomarker ratios (S2/S1) are represented by histograms (**center**) and spider plots (**right**). Participant E-044 is shown in the upper panel and participant E-078 is shown in the lower panel. The visualizations were created using Python libraries matplotlib (version 3.7.4), seaborn (version 0.13.2), and plotly (version 5.9.0). Histograms: red color: upregulation; blue: downregulation.

Supporting the observations of the pilot study, salivary biomarker ratio profiles showed clear associations with reported emotions at the individual level (Figure 3). For instance, participant E-044 displayed low/stable and slightly increased levels of sAA and OXT, respectively, with marked decreases in cortisol and DHEA following exposure to fragrances F2 and F3, a profile associated with an absence of emotional responsiveness. In contrast, the same participant exhibited a moderate decrease in sAA and moderate/high increased levels of cortisol, DHEA, and oxytocin when exposed to fragrances F4 and F5, corresponding to reports of moderate happiness and high sense of comfort and relaxation. Similar patterns were observed in other participants. For instance, E-078 displayed similar biomarker profiles for F2 and F4 characterized by elevated cortisol and DHEA levels with reduced oxytocin, aligning with relaxed emotional states and moderate happiness. Stimulation with F3 and F5 induced a different pattern, with decreases in all four biomarkers, that was associated with moderate happiness, relaxation, and confidence. These individual case studies illustrate how fragrance-related shifts in biomarker ratios parallel the individual’s emotional experience (Figure 3).

### 3.5. Biomarker Ratio Performance

We next applied multiple analytical approaches to examine biomarker dynamics across the entire study population: (i) principal component analysis (PCA) (Supplementary Figure S6), (ii) ANOVA (Supplementary Figure S7), (iii) hierarchical k-means clustering of biomarker ratio profiles (Supplementary Figures S8 and S9), and (iv) temporal clustering of baseline versus post-stimulation ratios (Supplementary Figure S10). Collectively, these analyses indicated associations between biomarker ratio profiles and reported emotional responses, although noise in the pooled dataset (all fragrances combined) limited the identification of clear BM–emotion pairings. Indeed, no significant differences between hk-means clusters were identified when emotional responses were discretized and assessed using Chi^2^ or PCA analyses (Supplementary Figure S11, Table S1).

### 3.6. Classification of Panelists’ Responses to Specific Fragrances

Because physiological and emotional responses to fragrances varied substantially across individuals and depended on the stimuli, we next performed individual-level clustering analyses for each fragrance. K-means and hk-means were applied to S2/S1 biomarker ratios (Supplementary Figure S12). For example, stimulation with fragrance F2 generated five clusters (n = 21, 22, 7, 9, and 1); in comparison, fragrance F5 produced four clusters (n = 28, 25, 3, and 3). The nested architectures obtained with fragrance F4 and F5 were different from those obtained with F2 and F3 fragrances. To explore hierarchical structure, hk-means was repeated with varying thresholds and recursively applied to subclusters.

**Figure 4:**
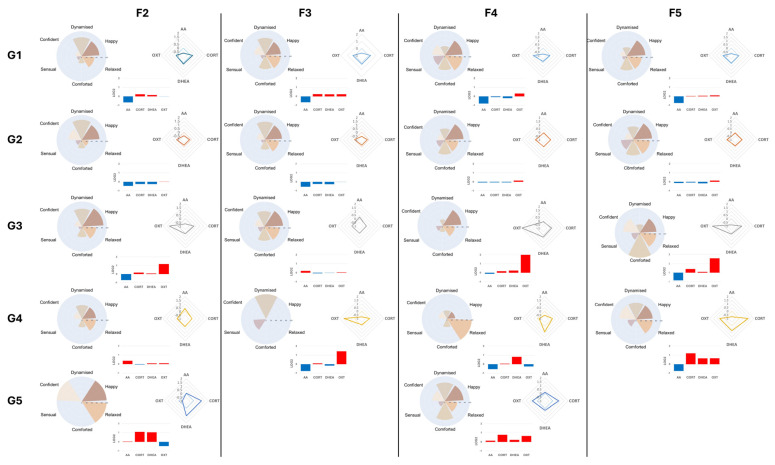
Emotional responses and salivary biomarker S2/S1 ratio profiles based on hk-means clusters by fragrance. For each fragrance (F2–F5), clusters are shown with emotional response and biomarker ratio profiles. The emotional response profiles are represented by a polar bar chart (**left**) that describes the % of participants per cluster who felt the emotion. Based on the estimated thresholds at 33% and 66% (dotted circles), emotional perception was categorized into three levels: low (<33%), moderate (33–66%), and high (>66%). Log2 mean biomarker ratios (S2/S1) are represented by histograms (**center**) and a radar plot (**right**). The baseline of the histogram and the dotted diamond on the radar plots represent the reference value (0). Red histograms above 0, in addition to log2 mean ratios above the dotted diamond, indicate upregulation; in comparison, blue histograms and log2 mean ratios below the dotted diamond indicate downregulation.

For each fragrance, biomarker distributions and emotional responses were systematically compared across clusters formed by hk-means (Figure 4). For each cluster, emotional responsiveness was classified as high (>66% reporting), moderate (>33% and <66%), or low (<33%). Using this framework, hk-means clusters exhibited distinct BM profiles that corresponded to specific emotion response profiles. For instance, clusters derived from fragrance F5 showed divergent combinations of happiness, relaxed, comforted, confident, and dynamised, each associated with a unique salivary biomarker profile. Strikingly, similar biomarker–emotion associations emerged across different fragrances. Clusters F4G2 (fragrance F4, group 2) and F5G2, including 24 and 25 individuals, respectively, both displayed modest decreases in levels of sAA, cortisol, and DHEA concomitant with moderately increased OXT levels, and participants in these clusters reported highly comparable emotional response profiles characterized predominantly by high happiness and moderate relaxation. Similarly, clusters F2G2 and F3G2 (21 and 25 individuals, respectively) were marked by pronounced decreases in levels of sAA, cortisol, and DHEA, with low decreased/stable levels of oxytocin corresponding to emotional response profiles centered on happiness and dynamism.

Together, these findings demonstrate that olfactory stimulations elicit distinct clusters of molecular and emotional responses within a population and that specific biomarker patterns can recur across different fragrances while maintaining consistent emotional associations. This conclusion suggests that clustering of salivary biomarkers could provide an objective framework for linking physiological responses to subjective emotional states. However, it is important to note that this is a proof-of-concept study with 59 participants exposed to each fragrance. Hierarchical k-means were performed on these subcohorts. As the clusters are formed, the number of individuals in each cluster decreases, which can weaken our statistical power. For example, Group 2 for Fragrances F2 and F3 consists of 21 and 25 individuals, respectively, while Groups for Fragrances F4 and F5 have 24 and 25 participants, and these clusters exhibit similar emotional profiles. Despite the limitations in cohort size, we observe that different fragrances can lead to distinct biomarker profiles associated with specific emotional states.

To further validate this approach, a larger cohort would be necessary to provide robust statistical analysis of the hk-means clustering results. Nonetheless, our current findings suggest preliminary evidence that salivary biomarkers clustered based on physiological responses can reliably indicate subjective emotional states.

### 3.7. Identification of Biomarker Profiles Related to Specific Emotions

To reduce the impact of the signal-to-noise ratio of biomarker measurements, data were discretized using the mean coefficient of variation in the biomarker assay (~10%) as the threshold. Ratios were categorized as decreased (<0.9), stable (0.9–1.1), or increased (>1.1), generating 81 possible combinations of BM states across 236 participant–fragrance exposures (Supplementary Figure S13).

**Figure 5:**
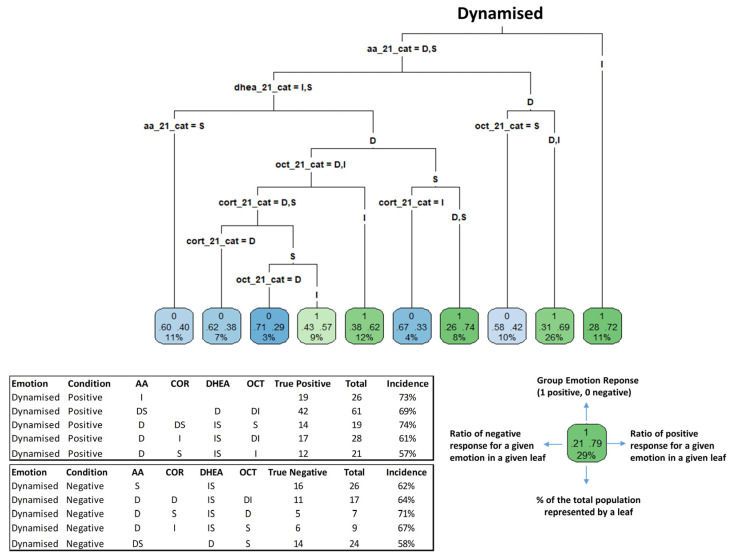
Classification of salivary biomarker ratio (S2/S1) profiles for dynamised emotion identification. The CART algorithm was employed using discrete variables of S2/S1 ratios of biomarkers. Participants were classified into the leaf categories based on whether they felt the emotion analyzed (positive: green) or not (negative: blue). The numbers and information shown inside the leaves are explained in further detail in the top right-hand corner of the figure. The intensity of the colour of the leaves is proportional to the ratio of response for a given emotion in a given leaf. The CART method was employed using the entire cohort data (236 unique combinations of individuals/fragrances).

**Figure 6:**
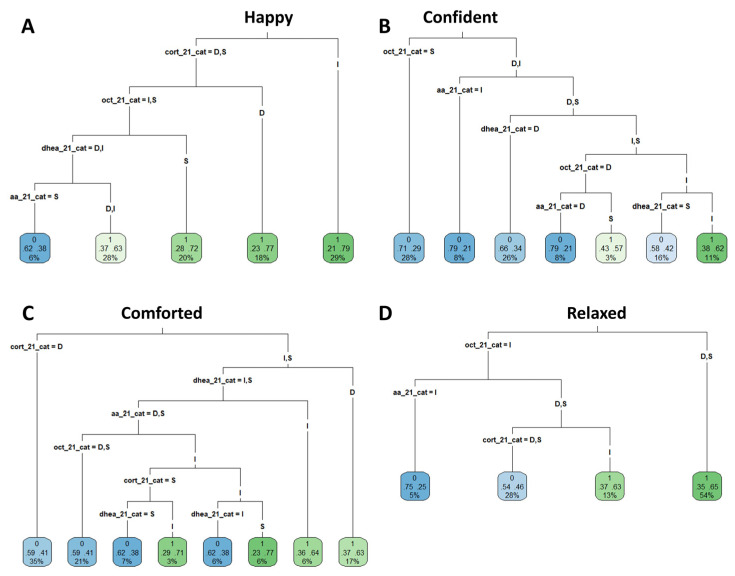
Classification of salivary biomarker ratio (S2/S1) profiles for emotion identification. The CART algorithm was employed using discrete variables of S2/S1 ratios of biomarkers. Participants were classified into the leaf categories based on whether they felt the emotion analyzed (positive: green) or not (negative: blue). The numbers and information shown inside leaves are explained in further detail in the top right-hand corner of the figure. The intensity of the colour of the leaves is proportional to the ratio of response for a given emotion in a given leaf. (**A**) CART for the happy emotion, (**B**) CART for the confident emotion, (**C**) CART for the comforted emotion, and (**D**) CART for the relaxed emotion. The CART method was employed using the entire cohort data (236 unique combinations of individuals/fragrances).

Classification and Regression Trees were subsequently used to predict emotional responses from biomarker profiles (Figure 5 and Figure 6). Using this approach, participants were classified into leaves, indicating either a positive (green) or negative (blue) response to a given emotion.

For the emotion “dynamised” (Figure 5), CART analysis resulted in the identification of distinct biomarker configurations: 69% of subjects with a profile of decreased/stable sAA levels, decreased DHEA levels, and variable OXT levels (decreased or increased) reported this emotion, representing 26% of the entire study population. Conversely, stable sAA levels combined with stable/increased DHEA was associated with the “dynamised less” feeling for 62% of the participants. Comparable CART decision trees were generated for the emotions happy, confident, comforted, and relaxed (Figure 6). For instance, 77% of participants with stable DHEA levels, increased cortisol and oxytocin levels, and decreased/stable sAA levels reported feeling comforted.

The accuracy of identifying different emotions using the biomarker signatures varied among emotions (Supplementary Table S2). Happy was identified with high sensitivity (97%) but low specificity (13%); in comparison, comforted and confident were predicted with high specificity (82% and 91%, respectively) but lower sensitivity (43% and 24%). These results indicate that discrete biomarker profiles capture emotion-specific molecular signatures, though predictive strength depends on the emotion assessed.

### 3.8. Emotional Profiles of Fragrances Based on Biomarker Profiles

**Figure 7:**
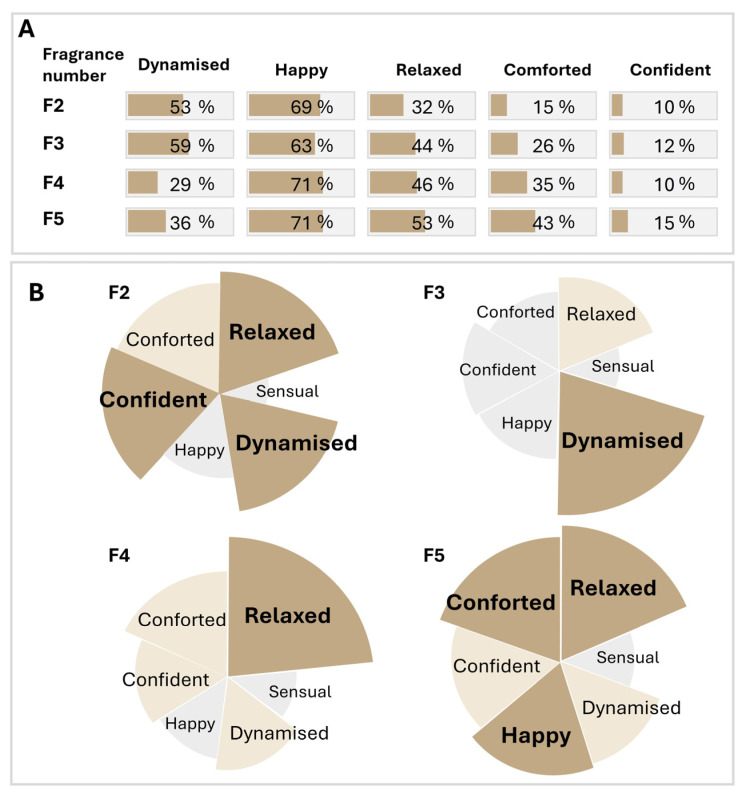
Biomarker-based emotional profiles induced by fragrances. (**A**) Emotional profiles induced by each fragrance (F2–F5) based on CART-derived salivary biomarker profiles. For each emotion, the percentage corresponds to the number of participants presenting the identified biomarker-based emotional profiles from the total number of participants that smelled the fragrance. The presented patterns are those identified from the decision trees generated based on CART analysis performed on the entire cohort data and biomarker ratios S2/S1 as discrete variables. (**B**) Implicit association test-based emotional profiles induced by each fragrance. Two parameters were measured with the implicit association test: the GO/NO GO-percent (the number of people who validated the emotion for each fragrance) and the strength of the association (the speed of response to validate the emotion for each fragrance). The GO/NO GO-percent is represented by the angle (emotions were presented 3 times during the test, and the % represents the number of clicks for each emotion). The strength of association (speed of clicks) is represented by the size and the color. An association is considered strong when rapid responses are given by the participant. Dark brown: excellent (>80 percentile, emotion shown in bold); brown: good (>50 percentile); gray: low (<50 percentile).

The CART-derived biomarker profiles were subsequently applied to characterize the emotional profiles induced by each fragrance (F2–F5). For each emotion, the proportion of participants with the corresponding biomarker ratio profile was calculated (Figure 7A). For example, the F5 fragrance elicited feelings of happiness, relaxation, and comfort; in comparison, the F3 fragrance evoked feelings of dynamism, relaxation, and happiness. Each fragrance elicited a distinct emotional signature, confirming that olfactory stimulation can be differentiated at the molecular level.

To validate these findings, an independent cohort (n = 80) completed an implicit association test (IAT) with the same four fragrances. The emotional profile derived from implicit measures (Figure 7B) produced similar results to those inferred from biomarker data. For example, for fragrance F3, at the level of the population, the biomarker-based emotional profiles and implicit association test-based emotional profiles appear to be similar. Indeed, the dynamised emotion is strongly felt, with 59% of the participants presenting the identified biomarker-based emotional profile and representing the higher score for the strength of association (23.26) and Go-percent (42.7%) measured using the IAT. The relaxed emotion was moderately felt, with 44% of participants presenting the biomarker-based emotional profile and moderate strength of association (17.34) and a relatively lower Go-percent (39.6%). The other emotions were only felt to a very slight degree by the participants (>30%), with the strength of association and Go-percent being lower. Only the profile for the emotion ‘happy’ varied differently for fragrances F2, F3 and F4. Similar results between the two approaches were observed for the other fragrances. All of these data were obtained through two complementary and orthogonal approaches, supporting the robustness of the biomarker-based approach.

### 3.9. Role of Individual Biomarkers in Fragrance Valuation

**Figure 8:**
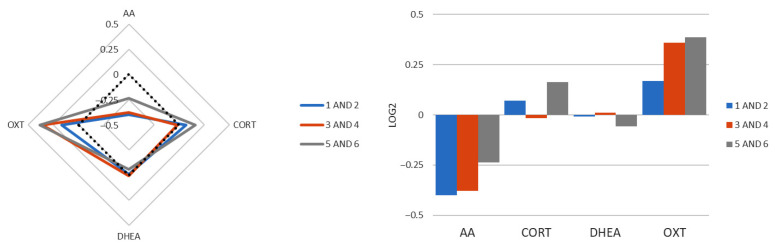
Salivary oxytocin and alpha-amylase ratio (S2/S1) levels associated with the valuation of all fragrances combined. In the questionnaire, each participant rated their valuation for each fragrance on a scale of 1 to 6: 5/6 corresponding to lower valuation; 3/4 to medium valuation; and 1/2 higher valuation. The radar plot describes the average levels of the ratio (S2/S1) for each biomarker according to the valuation groups (left panel). In the right-hand panel, histograms show the mean ratios of alpha-amylase and oxytocin ratios according to the fragrance valuation groups. This analysis was performed using data from the entire cohort (236 unique combinations of participants–fragrances).

Lastly, we examined whether individual biomarkers were associated with subjective fragrance valuation across the entire dataset (236 participant–fragrance combinations; Figure 8). Participants who rated fragrances poorly (scores 5–6) exhibited lower sAA ratios and higher oxytocin ratios compared with those giving medium (3–4) or high (1–2) ratings. These findings suggest that sAA and oxytocin may contribute to the evaluative dimension of fragrance perception, linking molecular changes not only to emotional states but also to hedonic valuation.

## 4. Discussion

This study provides a proof-of-concept that salivary biomarkers can be objectively used to characterize emotional responses to olfactory stimulation in real time. Using validated assays with high reproducibility, we quantified alpha-amylase, cortisol, dehydroepiandrosterone, and oxytocin levels in 888 saliva samples collected during controlled fragrance exposures. Our findings show that salivary molecular profiles can capture distinct physiological dimensions of emotion while avoiding the invasiveness or cognitive biases of self-report or neuroimaging [19,32].

*Multidimensional salivary profiling of olfactory emotion:* In previous olfactory–emotion studies, researchers have measured one biomarker, such as cortisol in odor-induced stress [11] or sAA changes during affective odor stimulation [33]. Our integration of four functionally distinct biomarkers captures broader dimensions of physiological spectrum and enables discrimination between affective states that may share valence or affiliative meaning but differ in arousal intensity. Weak inter-correlations among cortisol, alpha-amylase, DHEA, and oxytocin underscore their complementary roles: cortisol reflects HPA-axis activation [2], sAA rises within minutes following sympathetic activation [18,23], DHEA modulates glucocorticoid effects and resilience [24,25], and oxytocin supports affiliative signaling [27,28]. In stress physiology, two-biomarker combinations such as cortisol and sAA or DHEA–cortisol ratios have been used to stratify performance and anxiety responses [34,35,36]. In this work, we extend the multidimensional approach to the four-biomarker dimension to better decipher fragrance-evoked emotions.

*Individual molecular signatures in olfactory emotion:* In response to the same fragrance, individual participants clustered into distinct biomarker-based subgroups. This result is consistent with psychophysiological evidence of marked inter-individual variability in odor perception and affective responses [6,37]. The results of neuroimaging studies have similarly shown that individuals with comparable subjective ratings can display divergent neural activation patterns to identical odor stimuli [38], suggesting trait-like differences in central–peripheral coupling. We observed reproducible biomarker–emotion signatures across different fragrances, supporting the concept of stable molecular “emotional phenotypes”. This finding parallels the affective startle paradigm, in which eyeblink reflexes are potentiated during unpleasant stimuli and attenuated during pleasant ones, with stable inter-individual differences considered trait-like affective profiles [39]. By analogy, the reproducible biomarker–emotion signatures observed in this study may reflect consistent molecular phenotypes of olfactory emotional processing.

*Machine learning for physiological emotion classification:* The results of CART decision tree analyses showed that discrete biomarker ratios can be linked to specific emotional states with transparent, rule-based classification. Through CART analyses, we identified biomarker configurations that denote the presence or absence of emotions such as happiness, comfort, or confidence, with varying balances of sensitivity and specificity. This result supports earlier work in affective computing, wherein physiological signals such as heart rate variability or skin conductance classified emotional valence with >80% accuracy [40]. While happiness was detected with high sensitivity but low specificity, comfort and confidence were classified with high specificity but lower sensitivity. These patterns highlight both the promise and the current limitations of molecular prediction of such emotions. Importantly, the interpretability of decision trees is an advantage in biological contexts: rather than providing blind predictions, they reveal decision rules that can be mechanistically tested. For instance, combinations of decreased sAA with increased oxytocin were consistently associated with comforted states, and stable sAA and increased DHEA predicted the absence of dynamised emotion. Such rules may generate hypotheses regarding the pathways connecting olfactory input to peripheral endocrine responses.

*Cross-validation with implicit measures:* The concordance between our biomarker-based profiles and an independent implicit association test conducted in a separate cohort strengthens the reliability of our approach. The results of previous studies have demonstrated that implicit affective measures correlate with physiological stress markers, such as cortisol [41] and facial electromyogram [42]. In this line, we found that implicit and biomarker-derived profiles converged for fragrance-evoked emotions. However, the emotion “happy” is more involved in the emotional response profile defined by the biomarker-based profile than the implicit association test for three fragrances (F2, F3, and F4). The IAT measures implicit biases; in comparison, salivary biomarker measurement reflects the integrated body’s responses to stimuli. These two complementary approaches assess the same events from different perspectives and parameters that may explain the differences observed. This overall cross-method consistency suggests that salivary biomarkers provide an objective complement to behavioral measures in emotion research.

*Biomarkers and hedonic appraisal:* We also observed associations between biomarkers and fragrance valuation. Lower alpha-amylase ratios and higher oxytocin ratios were associated with reduced valuation, suggesting that these markers may encode not only evaluative but also emotional dimensions of olfactory experience. While oxytocin is frequently associated with positive valence and prosociality [27], context-dependent negative effects have been reported [43], and sympathetic downregulation may reflect disengagement [44]. Thus, biomarker responses appear to capture a complex interplay of arousal, affiliation, and appraisal, rather than mapping linearly to valence polarity.

*Perspectives in personalization:* The identification of stable molecular subgroups points toward potential personalization of sensory interventions. In clinical contexts, peripheral endocrine profiles have been shown to predict psychotherapy outcomes in Post-Traumatic Stress Disorder, with baseline cortisol metabolism, DHEA–cortisol ratios [45,46], and HPA-axis reactivity (via the cortisol awakening response) all stratifying treatment response [47]. Analogously, biomarker-defined phenotypes could be used to tailor fragrance design or therapeutic olfactory applications to individual emotional sensitivity profiles. More broadly, this work illustrates how molecular signatures may enable individualized models of emotion that integrate subjective and biological readouts.

## 5. Conclusions

The results of this study demonstrate that multi-biomarker salivary profiling is affordable, reproducible, and a simple non-invasive tool to assess olfactory-induced emotions. By combining molecular data with machine learning and through validation against implicit measures, we provide a robust and relevant framework for decoding emotional responses. Multidimensional molecular profiling captures both population and individual expression of affective physiology, offering a bridge between central sensory processing and subjective experience. This objective approach has potential applications in affective neuroscience, sensory marketing, and personalized therapeutic interventions and could be extended to other sensory domains where rapid, non-invasive emotion assessment is required.

**Limitations and future outlook:** While the results of our proof-of-concept study demonstrate the potential of using biomarker concentration changes to detect rapid emotional responses, further studies with larger sample sizes are necessary to assess repeatability and robustness. These future studies should include additional controls for confounding variables and more extensive statistical analyses to validate the predictive power of these biomarkers. Our biomarker panel does not currently include catecholamines or inflammatory mediators, which also respond dynamically to affective stimuli [48,49,50]. Other hormones or peptides present in saliva could also be included. Assay variability remains a challenge for some biomarkers such as salivary oxytocin. It is evident that larger and more diverse cohorts are required to increase the robustness of identified phenotypes. The current findings highlight the need to cluster individuals based on their emotional responses and biomarker profiles; through such measures, we can more effectively understand the variability within and between groups and develop a more refined framework for predicting emotional states using salivary biomarkers. This approach would provide a clearer picture of the consistency of these patterns across different populations and under various conditions. Lastly, integrating molecular profiling with concurrent neural and autonomic measures will be essential to map the temporal dynamics linking central olfactory processing to peripheral molecular responses.

## 6. Patents

Molina, F., Molina, L., Santos Schneider, F., Kahli, M. Method for identifying an emotion in a subject from saliva (PCT/EP2024/088625).

## Data Availability

The authors declare that all relevant data have been provided within the manuscript and its supporting information files.

## Supplementary Materials

The following supporting information can be downloaded at: https://www.mdpi.com/article/10.3390/bios16020081/s1, Figure S1: Stability of salivary biomarkers as a function of storage temperature for 48 hours; Figure S2: Salivary biomarker concentrations according to circadian rhythm; Figure S3: Example of individual representation of emotions and evolutionary profiles of biomarkers; Figure S4: K-means clustering of individuals, stimulated by F1 fragrance, into 6 groups based on biomarker profiles; Figure S5: Correlation heatmaps of salivary biomarker concentration ratios; Figure S6: Principal component analysis (PCA) of biomarkers ratios (S2/S1 and/or S3/S1) in relation to emotions; Figure S7: ANOVA analysis of salivary biomarker ratios among different groups of emotion valence (0 to 2) assessed in the questionnaire; Figure S8: Participants were classified based on salivary biomarker’s ratio profiles using hierarchical k-means (hk-means); Figure S9: Salivary biomarker profiles within clusters identified by hk-means; Figure S10: Temporal evolution of individual salivary biomarker ratios (S1/S1; S2/S1; S3/S1) in clusters identified by hk-means; Figure S11: Principal component analysis (PCA) was performed on discretized binary emotions and correlations to different variables were analyzed; Figure S12: Classification of participants based their salivary biomarker ratios (S2/S1) for each independent fragrance using hierarchical k-means (hk-means); Figure S13: Discretization of salivary biomarker ratios (S2/S1) for new clustering approaches; Table S1: Questionnaire-based emotional profiles in hk-means clusters; Table S2: Performances of salivary biomarker patterns for emotion identification.

## Abbreviations

The following abbreviations are used in this manuscript:

EEG	Electroencephalography
fMRI	Functional magnetic resonance imaging
HPA	Hypothalamic–pituitary–adrenal
ANS	Autonomic nervous system
CNS	Central nervous system
CRT	Cortisol
sAA	Salivary alpha-amylase
DHEA	Dehydroepiandrosterone
OXT	Oxytocin
BMs	Biomarkers
LDA	Linear Discriminant Analysis
RT	Room temperature
F0	Fragranceless support
F	Fragrance
PCA	Principal component analysis
hk-means	Hierarchical k-means
CART	Classification and Regression Trees
